# The Role of PLA2R in Primary Membranous Nephropathy: Do We Still Need a Kidney Biopsy?

**DOI:** 10.3390/genes14071343

**Published:** 2023-06-26

**Authors:** Thomas McDonnell, Henry H. L. Wu, Smeeta Sinha, Rajkumar Chinnadurai

**Affiliations:** 1 Department of Renal Medicine, Northern Care Alliance NHS Foundation Trust, Salford M6 8HD, UK; thomas.mcdonnell@nca.nhs.uk (T.M.); smeeta.sinha@nca.nhs.uk (S.S.); rajkumar.chinnadurai@nca.nhs.uk (R.C.); 2 Renal Research Laboratory, Kolling Institute of Medical Research, Royal North Shore Hospital, The University of Sydney, Sydney, NSW 2065, Australia; 3 Faculty of Biology, Medicine & Health, The University of Manchester, Manchester M1 7HR, UK

**Keywords:** phospholipase A2 receptor, antibodies against phospholipase A2 receptor, non-invasive diagnosis, prognosis, primary membranous nephropathy, secondary screening, kidney biopsy

## Abstract

Membranous nephropathy (MN) is the most prevalent cause of nephrotic syndrome amongst the non-diabetic adult population. A fifth of idiopathic nephrotic syndrome cases can be attributed to MN, rising to more than 40% in older patients over 60 years. Most MN cases are classified as being of a primary cause, where there is absence of a secondary disease process explaining its manifestation. Traditionally, the standard approach of diagnosing MN involves performing a kidney biopsy as histological evaluation offers not only conclusive evidence of the diagnosis but also provides valuable information regarding disease chronicity and the presence of any other kidney histopathological features. Nevertheless, kidney biopsy is an invasive procedure which poses risks for the patient including bleeding and pain and bears greater costs for the health system. The identification of the phospholipase A2 receptor (PLA2R) antigen in 2009 was a landmark discovery, one which has evolved our understanding of the disease processes in MN and subsequently our management approach of this condition. Antibodies against PLA2R (PLA2RAb) have since emerged as an attractive non-invasive test option to be applied for the diagnosis and prognostication of primary MN. However, much debate and unknowns remain about the accuracy and reliability of testing for PLA2RAb across various primary MN scenarios. We provide a review summarizing the historical journey of PLA2R in relation to its significance in primary MN and, more importantly, evidence emerging over the years which contemplated the role of PLA2RAb as a diagnostic and prognostic tool in primary MN.

## 1. Introduction: The Historical Evolvement of Defining Primary MN

Membranous nephropathy (MN) is the leading cause of nephrotic syndrome in non-diabetic adults. It is defined histologically by the thickening of the glomerular basement membrane due to deposition of sub-epithelial IgG deposits and has a classic ‘spiked’ appearance on silver staining [1]. In total, 20–25% of MN occurs due to a secondary cause. Causes include malignancy, autoimmune disorders, infections (classically hepatitis B), and medications (gold, penicillamine, and non-steroidal anti-inflammatory medications) [2]. Approximately 80% of MN occurs without a secondary disease process and is otherwise classified as primary MN. The incidence of primary MN is 1 case per 100,000 each year, with a 2:1 male: female ratio. It is responsible for over 20% of cases of idiopathic nephrotic syndrome, rising to over 40% amongst those over the age of 60 years [2,3].

Following the discovery of the m-type phospholipase A2 receptor (PLA2R) antigen by Beck et al. in 2009, there was a significant shift in the comprehension of primary MN etiology [4]. This work firmly established primary MN as a renal-limited autoimmune disease in which circulating autoantibodies attach to an autoantigen present on the podocytes’ surface resulting in glomerular protein leak. This discovery led to the shift in the definition of this phenomenon from ‘idiopathic’ to ‘primary’ MN. However, the concept of a permeability factor in MN is not novel. MN was first described as a pathologically distinct entity in 1957 by D.B. Jones [5]. A year later, German pathologist Ernst Walter Heymann published his landmark paper describing ‘Heymann’s nephritis’. Here, a mixture of rat kidney cell suspension and Freund’s adjuvant was injected into the peritoneum of healthy rats. These rats then developed a syndrome resembling human nephrotic syndrome, with histological changes similar to those seen in human glomerulonephritis, including the formation of immune complexes in the glomeruli. As the disease was specific to the particular kidney cell suspension used, Heymann concluded that the disease was due to an autoimmune reaction against an antigen in the glomerular epithelium. He named this antigen “Heymann antigen” [6]. Though this antigen was later shown to be the brush border protein megalin not present in human glomeruli, the concept of MN as an antibody-driven disease was nonetheless conceived from this discovery. Debiec et al.’s 2002 paper added further towards this narrative, where MN was identified in a fetus born to a mother congenitally deficient in neutral endopeptidase (NEP). A fetal kidney biopsy showed epithelial deposits of IgG and C3. It was postulated that maternal antibodies against NEP traversed the placenta to cause MN in the fetus at birth, supporting the hypothesis that MN is indeed an antibody-mediated disease [7]. Ultimately, it was Beck et al.’s discovery of the PLA2R antigen that redefined ‘idiopathic’ MN to ‘primary’ MN and solidified it as an antibody-driven disease.

## 2. PLA2R and Antibody-Mediated MN

In normal kidney tissue, PLA2R is highly expressed in glomerular podocytes, but its function remains largely unknown [8]. Antibodies directed against PLA2R specifically target the N-terminal region of the PLA2R protein, most commonly within the three most abundant N-terminal domains [9]. These antibodies predominantly belong to the IgG4 isotype and correspond to IgG4 immune deposits on kidney biopsy, with alternative IgG subclasses being more commonly seen in secondary MN biopsies [10]. A pathogenic model of PLA2R antibodies has recently been described, as evidenced by the induction of proteinuria and primary MN in a transgenic murine model using antibodies against PLA2R (PLA2RAb) IgG derived from rabbits [11].

In Beck et al.’s seminal study, the M-type PLA2R was identified as the target antigen in 70% of those with primary MN. Importantly, it was not found to be present in serum samples from patients with secondary MN or in those with other causes of nephrotic syndrome or healthy controls [4]. In the study, PLA2RAb levels correlated with disease activity, and falling levels predicted remission and reduced urinary protein excretion. Further studies have validated utilizing PLA2RAb as a serum biomarker of disease activity. Its routine measurement can be used to predict disease activity and relapse [12], as well as predicting remission, either through low presenting levels predicting spontaneous remission [13] or persisting positivity after immunosuppression conferring a negative outcome [14].

Not only have PLA2RAb levels been shown to guide response to treatment, but the identification of a specific target antigen has helped to change the management of MN. Targeted therapy aimed at reducing antibody production has been evaluated in randomized control trials. Rituximab, a B-cell-depleting therapy, was compared to non-immunosuppressive anti-proteinuric therapy. The trial demonstrated that rituximab was superior in reducing PLA2RAb levels and induced remission more frequently [15]. Subsequently, the MENTOR trial compared rituximab therapy to cyclosporine and found that although the therapeutic remission rates were similar, the decline in PLA2RAb levels was faster, greater in magnitude, and of longer duration in the rituximab group [16]. These findings have led to the recommendation of the modified Ponticelli regimen or rituximab therapy in PLA2R-positive MN and demonstrated how identifying the target antigen has altered the management of MN [17].

Beck et al. initially reported that 70% of their primary MN cohort was PLA2R-positive, despite this being a relatively small cohort of just 36 primary MN patients [4]. This result has been shown to be consistent with a number of studies which were conducted at a later time period [13,18]. Nevertheless, this proportion still leaves a gap in the diagnostic algorithm. There would be 25–30% of MN patients with no identifiable secondary disease who appear to be PLA2R-negative. Following the inaugural antigen discovery, there have been a number of novel antigens reported in association with MN. It may well be that the continuous discovery of novel antigens may further reclassify primary MN to a more sophisticated model of ‘antibody mediated’ MN. Brief descriptions of these novel antigens discovered over the past three decades are presented here in Table 1.

There is no doubt that the emergence of PLA2RAb has changed the way we understand primary MN. Its etiology as an antibody-driven glomerulopathy has been solidified, and PLA2RAb testing has become conventional for the workup, monitoring, and to help guide clinical decision making. Yet despite a number of studies detailing its impressive characteristics as a non-invasive diagnostic test option, routinely referring patients who are PLA2R-positive for kidney biopsy has remained a popular approach amongst nephrologists [29]. Despite the potential advantages of avoiding a kidney biopsy, including logistical and safety benefits, many nephrologists have not embraced utilizing PLA2R tests as the sole diagnostic tool in routine clinical practice. We examine in further detail whether a kidney biopsy is still warranted in cases of PLA2R-positive membranous nephropathy, given the availability of routine PLA2RAb testing.

## 3. Traditional Workup for Primary MN

MN is traditionally associated with the clinical presentation of nephrotic syndrome, but its presentation is actually quite heterogeneous. In a case series involving 116 patients diagnosed with primary MN, although the majority demonstrated nephrotic range proteinuria (>3 g/24 h), a quarter of individuals exhibited sub-nephrotic proteinuria. Furthermore, 55% of the patients presented with microscopic hematuria [30]. After thorough evaluation of patient history, meticulous physical examination, and exclusion of secondary causes, the standard and prevailing approach remains to perform a kidney biopsy. When an adequate sample is obtained, this procedure not only offers conclusive evidence of the diagnosis but also provides valuable information regarding disease chronicity and the presence of any alternative diagnoses.

Performing a kidney biopsy is an invasive procedure and not without its risks. A meta-analysis published in 2020 of 118,064 cases presented results showing patients presenting with pain (4.3%), hematoma (11%), macroscopic hematuria (3.5%), indication for blood transfusion (1.6%), procedural intervention to stop bleeding (0.3%) (approximately 1 in 300), and death (0.06%) (approximately 1 in 1500) following kidney biopsy [31]. A retrospective review of ultrasound-guided kidney biopsies over a 20-year period concluded an overall rate of complications of 16.6%, of which 1.5% were defined as being major complications [32], with another study showing the rates of complications increasing further when biopsies were performed in an emergency setting [33].

The cost of performing kidney biopsies should be factored in as well. In the UK, the cost of performing a kidney biopsy is almost 30 times that of undertaking a PLA2R antibody test, not considering the cost of complications or the societal cost of time off work for the patient [34]. In the United States, the cost of performing a kidney biopsy for those without insurance coverage is profound at an estimated USD 10,000 [35]. There is also the additional time cost that a kidney biopsy imparts. For instance, a 24 h post-biopsy observation is sometimes required, which would necessitate an overnight hospital stay.

It is evident that the inclusion of a specific non-invasive biomarker such as PLA2RAb would be highly beneficial in addressing issues related to safety, cost, and time constraints. However, in order to rely solely on this biomarker, it would have to offer the same level of diagnostic and prognostic value to what a kidney biopsy can provide.

## 4. Summary of Evidence Evaluating PLA2RAb as a Diagnostic Tool for Primary MN

A meta-analysis published in 2014 examined the sensitivity and specificity of PLA2RAb as a diagnostic marker for primary MN [29]. Nine studies were included encompassing 2212 patients—four were prospective studies, and five were retrospective studies. There were three studies which did not have controls and three which only included other kidney disease patients as controls. The pooled sensitivity was 78% (95% CI: 66% to 87%) and the specificity was 99% (95% CI: 96% to 100%). The positive likelihood ratios were 96.1 (95% CI, 19.5 to 472.1) with a negative likelihood ratio of 0.22 (95% CI: 0.14 to 0.35). However, the I_2_ score was 93.7% for the summary sensitivity and 93.8% for the summary specificity, suggesting a high heterogeneity in this sample of studies. When comparing the included sensitivities and specificities across the individual studies, these ranged from 64.3% to 96.5% and from 33.3% to 100%, respectively (the two lower numbers, i.e., sensitivity 64.3% and specificity 33.3%, originated from the same study by Svobodova et al. [36], which was a small study with 31 patients. When sensitivity analysis was performed to assess the effect of the study on the pooled accuracy of PLA2RAb testing, it had a minimal effect).

Although the results of this meta-analysis were encouraging, as the authors acknowledged, the studies included in this meta-analysis were generally of modest methodological quality. There was also limited information as to the timing of biopsy in relation to PLA2RAb testing. This is important as PLA2RAb titers vary depending on not only the timeline of disease progression but also on immunosuppression and immunological remission status subsequently. There is also heterogeneity regarding the type of test that was undertaken to measure PLA2RAbs. The immunofluorescence assay (IFA), enzyme-linked immunosorbent assay (ELISA), and (non-commercially available) Western blot were all used, suggesting results were less translatable to the clinical practice setting.

The hesitancy of nephrologists to adopt PLAR2Ab testing as the sole diagnostic test for primary MN is not merely due to issues such as limited sample sizes or inadequate methodological validity from studies conducted. Rather, the key question of whether a biopsy is still necessary remains unanswered. Given a low test sensitivity, it is clear that in the presence of a negative PLA2RAb, a biopsy would still be needed. However, one would think the specificity of nearly 100% would obviate the need for further investigation with kidney biopsy in the context of a positive PLA2RAb. Crucially though, the individual studies did not define in what patient cohort a positive test alone was sufficient and, critically, if other diagnoses besides primary MN would be overlooked by foregoing biopsy. Secondly, it remained unclear whether the omission of kidney biopsy would result in the loss of prognostic data. There were three published studies which aimed to address this specific question.

In a retrospective study published in 2019, Bobart et al. [37] reviewed all PLA2RAb results in 838 patients from one center, where 143 patients had a positive PLA2RAb test by combined ELISA and IFA. In total, 132 had a native renal biopsy result and 35 were excluded due to ‘secondary’ causes of MN. ‘Secondary’ causes were documented as being due to autoimmunity (eleven patients), positive chest X-ray findings suggestive of lung cancer (two patients), positive prostate-specific antigen test results suggestive of prostate cancer (four patients), medication-associated causes (three patients), paraproteinemia (six patients) and other malignancies (nine patients). In regard to other malignancies, Bobart and colleagues did not state if these were already known malignancies or if they were found during a screening process. If it was the latter, the screening process has not been detailed by the investigators. Of the remaining 97 patients, all had histology consistent with primary MN, but 7 patients had an additional diagnosis found on kidney biopsy. The authors then split the cohort into those with an estimated glomerular filtration rate (eGFR) above and below 60 mL/min/1.73 m^2^. Amongst those with an eGFR > 60 mL/min/1.73 m^2^, two patients had additional diagnoses—one patient had a superimposed focal segmental glomerulosclerosis (FSGS) lesion and the other had a superimposed diabetic nephropathy, neither of which the authors felt had altered the patient’s clinical management. The mean Total Renal Chronicity Score (TRCS), a histopathological grading system of severity, was 0.4. Amongst those patients with an eGFR < 60 mL/min/1.73 m^2^, five patients had additional diagnoses—one patient had acute interstitial nephritis, one had superimposed diabetic nephropathy, one had a superimposed acute tubular necrosis, and two patients had superimposed FSGS lesions, one of which also had cellular crescent. The mean TRCS in this group was 1.0. From this study, it appeared those with PLA2RAb positivity and an eGFR > 60 mL/min/1.73 m^2^ did not have any additional diagnoses seen on biopsy that would alter clinical management, though the addition of diabetic nephropathy on biopsy could confound the degree of proteinuria attributed to MN. The eGFR correlated well with chronicity scores on biopsy, thus kidney biopsy did not provide any additional prognostic data, particularly as the significance of an FSGS lesion in those with preserved renal function is unknown [38]. In those with an eGFR < 60 mL/min/1.73 m^2^, two of the five additional diagnoses would change clinical management (i.e., interstitial nephritis and FSGS with crescents), suggesting that kidney biopsy may still be required in those with eGFR < 60 mL/min/1.73 m^2^. Ultimately, eGFR correlated well with chronicity scores, which indicates that kidney biopsy was needed for prognostication in the low eGFR cohort.

In another study published in 2019, Wiech et al. [39] evaluated 263 patients with a histological diagnosis of MN. The authors stated secondary causes were excluded, though there were no details on how the exclusion process was undertaken. The authors retrospectively compared histological results to the measured PLA2RAb levels. This was an important difference from Bobart et al.’s approach, in which they commenced their study with positive PLA2RAb cases and worked towards investigating the corresponding histology. The approach by Wiech et al. is open to verification bias as they selected only those patients who have already been diagnosed with primary MN. In this case, PLA2RAb is used to confirm the diagnosis rather than to establish it, which can lead to an overestimation of sensitivity and specificity of PLA2RAb. PLA2RAb was detected in 194 patients (74%). Twelve patients (6%) had an additional diagnosis on kidney biopsy, in which five patients were diagnosed with diabetic nephropathy, five patients were diagnosed with interstitial nephritis, and two patients were diagnosed with IgA nephropathy. The latter two would likely alter management, and again the presence of diabetic nephropathy may confound proteinuria, as detailed above. Though there was no breakdown of each individual’s eGFR results, those with a second diagnosis had a median eGFR of 51 mL/min/1.73 m^2^ (29–72) compared to 84 mL/min/1.73 m^2^ (50–101) in those without a second diagnosis, fitting in with Bobart et al.’s cut off of eGFR < 60 mLs/min/1.73 m^2^.

Bobart et al. then revaluated the utility of PLA2RAb in a validation study in 2021 [40]. PLA2RAb tests were ordered in 2313 patients, of which 163 patients had a positive PLA2RAb test and native kidney biopsy result. Forty-seven patients were excluded due to secondary disease, listed as being due to autoimmune disease (seventeen patients), malignancy (ten patients), non-steroidal anti-inflammatory drug use (seven patients), hepatitis (six patients), paraproteinemia (six patients), or a combination of paraproteinemia and malignancy (one patient). The process of screening for malignancy was not detailed. In this validation cohort, patients were also excluded if they had diabetes (15 patients), leaving 101 patients in total for evaluation. All 101 participants had primary MN on biopsy. In total, 12 of the 101 patients (12%) had an additional diagnosis. When splitting patient groups by eGFR, there were seven patients with secondary diagnoses identified in kidney biopsy amongst those with eGFR > 60 mLs/min/1.73 m^2^—one FSGS lesion, one focal GBM disease, three capillary loop fibrin thrombi, and two acute tubular injuries. None of these secondary diagnoses were felt to alter clinical management or prognosis. In the group with eGFR < 60 mLs/min/1.73 m^2^, five patients had secondary diagnoses—two FSGS lesions, two acute tubular injury, and one interstitial nephritis. TRCS again correlated well with eGFR and kidney biopsy findings and did not provide additional prognostication value for those with an eGFR > 60 mLs/min/1.73 m^2^.

## 5. The Significance of an FSGS Diagnosis on Kidney Biopsy

The presence of an FSGS lesion on kidney biopsy was seen in both of Bobart et al.’s cohorts from the 2019 and 2021 publications, and it would be important to appreciate the significance of this finding. One study examining the association between FSGS lesions and concurrent MN depicted that this association was more likely to present in patients with lower creatinine clearance and those who receive anti-proteinuric medication. The amount of proteinuria, the likelihood of receiving immunosuppression, and more importantly clinical outcomes, including decline in kidney function or response to treatment, were the same between MN patients with and without FSGS as identified from biopsy [38]. This suggests that the additional outcome of FSGS lesions confirmed histologically should not change the direction of clinical management, and this finding does not outweigh the negatives associated with performing routine kidney biopsy.

## 6. Should We Biopsy the Diabetic Patients Suspected with MN?

From the above studies, it remains unclear if all diabetic patients should be referred for kidney biopsy to allow for primary MN workup. If a diabetic patient presents with nephrotic syndrome or nephrotic range proteinuria, has secondary causes excluded, and is PLA2Rab-positive, it remains unclear how much of their proteinuria can be attributed to MN or diabetic nephropathy and if there is an alternative undiagnosed pathology. In a study examining 620 kidney biopsy results of diabetic patients, 37% of patients had diabetic nephropathy, with 36% of patients diagnosed with non-diabetic kidney disease and 27% of the patients having features of both diabetic nephropathy and non-diabetic kidney disease. In total, 80% of the non-diabetic kidney disease diagnoses were found to be FSGS (22%), hypertensive nephrosclerosis (18%), acute tubular necrosis (17%), IgA nephropathy (11%), membranous glomerulonephritis (8%), and pauci-immune glomerulonephritis (7%) [41]. This emphasizes the broad spectrum of kidney pathologies that those with diabetes can have and the difficulty in determining these pathologies amongst diabetics. As a consequence, Bobart and colleagues excluded diabetic individuals from their second validation study, with awareness that this weakened the conclusions which could be drawn from this context.

## 7. Utility of Glomerular PLA2R Staining with Undetectable Serum PLA2RAb

As described previously, PLA2RAb could be utilized to predict immunological response in primary MN, and thus it would be important to consider the timing of antibody measurement in relation to the disease course to ensure accurate readings as much as possible. There are two scenarios in which a patient with primary MN would display seronegative PLA2RAb. Firstly, the patient may be in remission—this is either spontaneous or secondary to immunosuppressive medication. Otherwise, it may be too premature to detect PLA2RAb in the serum, as a consequence of the ‘immunological sink’ phenomenon. In both situations, glomerular PLA2R staining could be of use.

In the former scenario, if PLA2RAb cannot be detected and secondary causes have been excluded, glomerular PLA2R staining can act as the footprint for previous immune activity. This is evidenced from the retrospective study by Svobodova et al. [36] reviewing historic primary MN biopsy samples, where 22% sera sampled at the time of remission were PLA2R-positive, while 59% were positive for glomerular PLA2R staining.

In the latter scenario, which usually occurs early in the disease course, PLA2RAb is produced and binds to the PLA2R antigen on the podocyte and is cleared from the bloodstream faster than it is produced. In this situation, the kidneys act as an ‘immunological sink’. Only later when the antigens become saturated will serum levels rise towards detectable levels. This is where glomerular staining will be positive for PLA2R and the diagnosis of primary MN can be determined [42].

## 8. The Different Methods of Testing for PLA2RAb

Based on the above referenced studies, current guidance from international study groups such as Kidney Disease Improving Global Outcomes (KDIGO) favor and recommend that a kidney biopsy would not be necessary in PLA2Rab-positive MN patients with normal kidney function (though it does encourage the clinician to consider performing a biopsy if immunosuppression is being administered) [17]. It is of vital importance, however, that the clinician understands what a positive PLA2RAb test is and is aware of the testing methods employed by their laboratory. If they are transferring the implications of results from the studies that were previously mentioned, the clinician needs to understand if these results correlate with findings within the context of their local clinical practice.

As mentioned in the aforementioned meta-analysis, there are three distinct methods for measuring PLA2R. The first method, based on ELISA, employs recombinant human PLA2R antigen to quantitatively measure the levels of PLA2R IgG, IgA, and IgM in patient serum, typically reported in relative units (RU) per milliliter [29]. The threshold for a “positive test” can range from 10 to 40 RU/mL depending on the laboratory. The second method, based on IFA, utilizes cells transfected with the PLA2R antigen to measure PLA2R IgG levels in patient serum, expressed in semi-quantitative dilutional titers (<1:10, 1:10, 1:20, 1:40). IFA is considered more specific in comparison to ELISA. Lastly, there is Western blot analysis, which was the original test used by Beck et al. [4]. This is the most specific but non-commercially available method for measuring PLA2R antigen. Such operational differences must be considered when interpreting PLA2RAb results in clinical practice.

In their study published earlier this year, Ragy et al. [34] highlighted the importance of understanding the characteristics of a diagnostic test and how one’s own laboratory processes PLA2RAb results. In their study, all of the patients with positive PLA2RAb test results determined by ELISA and native kidney biopsy over a 17-year period were included. Of the 187 patients analyzed, 73 patients (39%) had a positive PLA2RAb, and 94 patients (50.2%) had a diagnosis of primary MN. Furthermore, 71 of the 94 primary MN patients (75.5%) tested positive for PLA2R. No cases of secondary MN were associated with a positive PLA2RAb. The anti-PLA2R ELISA had a specificity of 0.98 and a sensitivity of 0.76, with a positive predictive value (PPV) of 97.3% and a negative predictive value (NPV) of 79.8%.

However, the study identified two patients without primary MN on biopsy who tested positive via ELISA. These tests were both only weakly positive at 31 RU/mL and 36 RU/mL. This underscores the fragility and artificial nature of attributing a binary interpretation to a positive test in a quantitative assay. In Bayesian analysis, a lower titer holds less significance compared to the higher titer. If we lower the positive threshold to define disease, there would be increased sensitivity but reduced specificity, and increasing the positive threshold would result in the opposite.

When Ragy and colleagues lowered the ‘positive’ value of the ELISA test to >10 RU/mL in their study, it resulted in the highest value of Youden’s J statistic (this is a single number summary of the performance of a diagnostic test that considers both sensitivity and specificity), but this would have lowered the specificity of the test. The specificity of the test was highest when the positive titer was >40 RU/mL. These findings highlight the importance of considering the purpose of this test. The benefit of PLA2RAb is in a ‘positive’ result, as a negative result will indicate a referral for kidney biopsy, whereas the benefit of a positive result is to potentially forgo biopsy. Clinicians would want to ensure all positive results are true positives (to avoid missing a diagnosis), and this is achieved by raising the positive threshold of a quantitative test and increasing its specificity. Although this approach may result in performing biopsies for extra cases of primary MN, this approach also provides reassurance that alternative diagnoses will not be missed.

In Bobart et al.’s study published in 2019 [37], results from ELISA and IF testing were combined in its interpretation. In this study, 110 patients had an ELISA value ≥ 2 RU/mL but ≤20 RU/mL (weakly positive). These patients then had confirmatory IFA testing. Thirty patients were positive on IFA and all had primary MN diagnosed in kidney biopsy. Eighty patients had a negative IFA result, in which forty-six patients (57.5%) had MN diagnosed on biopsy. Patients initially found to have weakly positive ELISA levels and negative IFA did not perform outwardly different than those with double negative testing (ELISA < 2 RU/mL and IFA negative), with 49.6% of patients found to have MN on biopsy. These results suggest that the reference value of the ELISA test could be lowered to ≥2 RU/mL if there is access to confirmatory IF testing from the laboratory. The 2021 study from Bobart et al. [40] included four separate sites with different reporting procedures for positive MN testing—two sites incorporating combined ELISA ≥ 2 RU/mL and confirmatory IF testing results, one site reporting with either ELISA ≥ 20 RU/mL or positive IF testing, and one only reporting when ELISA ≥ 20 RU/mL. Though specifics regarding individual values were not provided, there were no reported false positives.

There have been previous descriptions of false-positive PLA2RAb results within the published literature, though these have been largely anecdotal. A case report of a 74-year-old diabetic male with no evidence of MN on kidney biopsy had a positive anti-PLA2R ELISA test level of 217 RU/mL but negative IFA results [43]. This was further corroborated with reports of two further diabetic patients with positive ELISA of 85.7 and 182.9 RU/mL having no evidence of MN on biopsy, and again both displayed negative IFA results [44]. Though such findings are sparse, there does seem to be unexplained false-positive ELISA results amongst diabetic individuals, and if IFA testing is not available, these findings should lead the clinician to interpret a positive ELISA result in diabetic individuals with greater caution.

An increased incidence of false positivity in ELISA and IFA results has been reported in patients with secondary MN attributed to hepatitis B virus (HBV) [45]. This phenomenon, though not yet elucidated, underscores the significance of interpreting PLA2RAb within the framework of excluding secondary etiologies and emphasizes the importance of secondary screening up front, though of course these could represent disease associations which are non-causal. Furthermore, there are additional costs associated with investigating for secondary causes of disease, and there is a potential case to be made to avoid this process if PLA2RAb is positive nevertheless [46].

Furthermore, it is crucial to acknowledge that PLA2RAb testing has been validated primarily in European and North American Caucasian populations. A pertinent abstract presented by Mok and Choo at the 2020 European Renal Association—European Dialysis and Transplant Association virtual meeting [47] shed light on this issue. The abstract documented 31 patients of Chinese or Indian ethnicity with non-MN lesions observed on kidney biopsy, though these patients exhibited positive anti-PLA2R ELISA tests. Among these patients, twenty-six displayed ELISA levels below 50 RU/mL, while five patients demonstrated levels exceeding 50 RU/mL (ranging from 56 to 168 RU/mL). Notably, no IFA results were reported. Nevertheless, this underscores the necessity for further validation studies in non-Caucasian cohorts.

## 9. Considerations for Secondary Screening in MN

In all of the studies which examined PLA2RAb as a diagnostic tool for primary MN, those with secondary MN were excluded. It may therefore be clinically appropriate that this pathway is followed in practice to ensure patients match with the intended study population for which PLA2RAb is applied to. The major dilemma, however, is that the details of secondary screening employed within individual studies are inconsistently described. Bobart et al. [37,40] stated that ‘full laboratory and radiologic evaluation for secondary causes’ were undertaken; however, the specifics of results from these evaluations were not explicitly defined. KDIGO endorses the evaluation for secondary causes regardless of the PLA2RAb result [17]. It is recommended that HBV, hepatitis C virus, and human immunodeficiency virus screening is conducted, as well as enquiring for non-steroidal anti-inflammatory drug use. A full connective tissue disease medical history is also recommended, in addition to obtaining samples for antinuclear antibody testing and performing a chest X-ray to screen for sarcoidosis. Despite no mention in the KDIGO guidelines, clinicians should consider warranting routine serum protein electrophoresis measurements to be performed, given the substantial prevalence of paraproteinemia observed in the Bobart et al. cohorts. What remains less clear is regarding malignancy screening—in particular which malignancies should be screened for. KDIGO acknowledges the challenge in clinical judgment in this respect and notes that clinical practices vary by site and country. KDIGO recommends clinicians to adopt a routine screening strategy which is ‘population and age appropriate’ for their patient. Taking an adult male above age 50, this may include a chest X-ray or computed tomography scan, investigation for iron deficiency, participation in the national screening program for colon cancer, and a prostate-specific antigen (PSA) test. A review article published by Plaisier and Ronco on the subject of cancer screening in primary glomerulopathies advocates for an age-appropriate cancer screening approach but, in addition, advises clinicians to offer a more targeted diagnostic approach based on pre-morbid factors [48].

## 10. Conclusions

**Figure 1:**
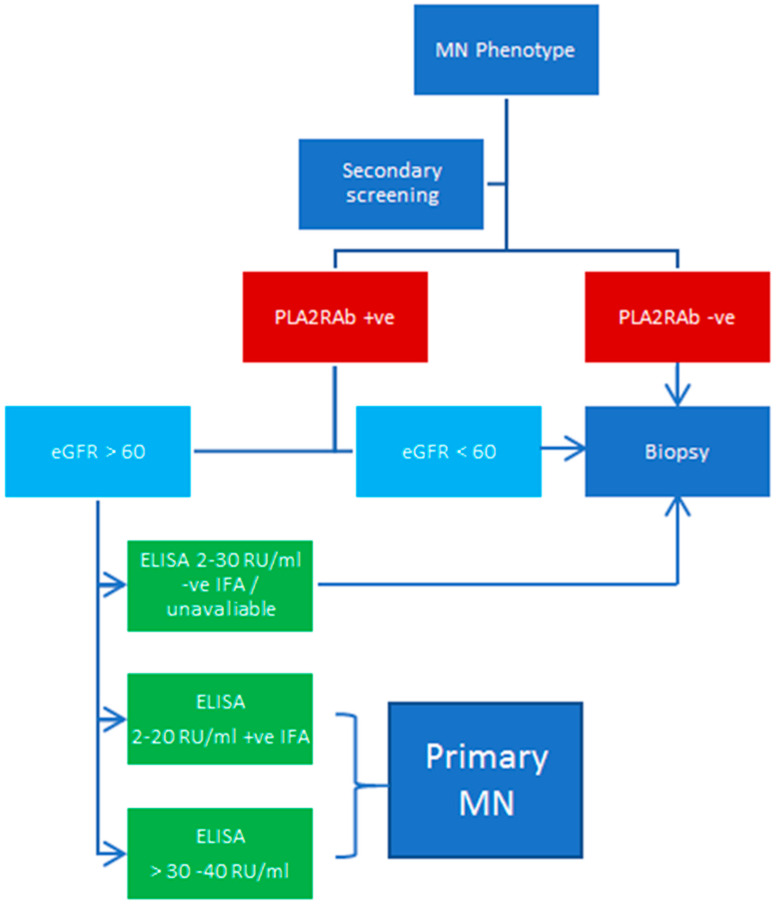
A proposed diagnostic approach for primary MN. eGFR: estimated glomerular filtration rate; ELISA: enzyme-linked immunosorbent assay; IFA: immunofluorescence assay; MN: membranous nephropathy; PLA2RAb: antibodies against phospholipase A2 receptor.

Identification of the PLA2R antigen since 2009 has revolutionized our understanding and management of primary MN. This discovery has not only transformed our comprehension of the underlying pathophysiological processes involved but also influenced our approach to the diagnosis, treatment, and follow-up of primary MN. Furthermore, recent studies have challenged the long-held belief that a kidney biopsy is necessary for the diagnosis of primary MN. PLA2RAb has reliably been seen only in cases of primary MN and has an approximate sensitivity and specificity of 75% and 100%, respectively. Though it is clear that a kidney biopsy is needed in the case of negative PLA2RAb, a biopsy may not be necessary in the context of a positive result. Studies have reliably shown that if eGFR is >60 mLs/min/1.73 m^2^, then there is no additional diagnosis seen on biopsy that would alter clinical management and no added information gained regarding chronicity or prognosis. It is important that a robust secondary screening process is undertaken prior to interpreting the PLA2RAb result in context. Careful interpretation of these results is required in diabetic patients. Lastly, it is extremely important to understand how these tests are performed and processed within local clinical practice. If only the ELISA testing is available, a positive result should be >30–40 RU/mL to ensure that there are no included false positives as much as possible. If the laboratory has access to IFA, then an ELISA test between 2 and 30 RU/mL with confirmatory IFA testing is sufficient to exclude false positives. Figure 1 summarizes our proposed approach to the diagnosis of primary MN non-invasively via PLA2RAb where possible, based on currently available evidence. As associated antigens in primary MN continue to emerge, it is anticipated that nephrologists will continuously engage in a more sophisticated approach when diagnosing primary MN and prognosticating disease progression for years to come.

## Figures and Tables

**Table 1:** Novel antigens associated with primary and secondary MN.

Antigen	Association to MN
Neural epidermal growth factor-like 1 (NELL1)	Reported in approximately 16% of primary MN [19]. Reports of associated malignancy [20].
Thrombospondin type-1 domain-containing 7A (THSD7A)	Reported in approximately 3% of patients with primary MN, some double positivity with PLA2R [21]. Increased rates of associated malignancy 20–50% [22,23].
Exostosin 1 and 2 (EXT1/EXT2)	Commonly seen in autoimmune/lupus-associated MN [24]
Protocadherin 7 (PCDH7)	Potential association with autoimmune conditions (SLE and Sjögren’s syndrome) and malignancy [25]
Semaphorin 3B (Sema3B)	More commonly seen in children/young adults, with possibly an inheritable component [26].
Serine protease high-temperature requirement A1 (HTRA1)	Potential association with malignancy and ANCA vasculitis [27]
Neutral endopeptidase (NEP)	Rare antenatal MN which can develop in the fetus of NEP-deficient mothers [7,28]

ANCA: antineutrophil cytoplasmic antibody; MN: membranous nephropathy; PLA2R: phospholipase A2 receptor; SLE: systemic lupus erythematous.

## Data Availability

Not applicable.
